# The chain of survival in hypothermic circulatory arrest: encouraging preliminary results when using early identification, risk stratification and extracorporeal rewarming

**DOI:** 10.1186/s13049-016-0281-9

**Published:** 2016-06-29

**Authors:** Tomasz Darocha, Sylweriusz Kosiński, Anna Jarosz, Dorota Sobczyk, Robert Gałązkowski, Jacek Piątek, Janusz Konstany-Kalandyk, Rafał Drwiła

**Affiliations:** Department of Anesthesiology and Intensive Care, the John Paul II Hospital, Medical College of Jagiellonian University, Cracow, Poland; Polish Medical Air Rescue, Warsaw, Poland; Department of Anesthesiology and Intensive Care, Pulmonary Hospital, Zakopane, Poland; Tatra Mountain Rescue Service, Zakopane, Poland; Department of Interventional Cardiology, the John Paul II Hospital, Cracow, Poland; Department of Emergency Medical Services, Medical University of Warsaw, Warsaw, Poland; Department of Cardiovascular Surgery and Transplantology, Collegium Medicum, Jagiellonian University, the John Paul II Hospital, Cracow, Poland

**Keywords:** Accidental hypothermia, Cardiac arrest, Cardiopulmonary resuscitation, Extracorporeal membrane oxygenation

## Abstract

**Background:**

The prognosis in hypothermic cardiac arrest is frequently good despite prolonged period of hypoperfusion and cardiopulmonary resuscitation. Apart from protective effect of hypothermia itself established protocols of treatment and novel rewarming techniques may influence on outcome.

The purpose of the study was to assess the outcome of patients with hypothermic circulatory arrest treated by means of arterio-venous extracorporeal membrane oxygenation (ECMO) according to locally elaborated protocol in Severe Accidental Hypothermia Center in Cracow, Poland.

**Methods:**

Prospective observational case-series study – all patients with confirmed hypothermic cardiac arrest consulted with hypothermia coordinator were accepted for extracorporeal rewarming, unless contraindications for ECMO were observed (active bleeding).

**Results:**

The study population consisted of 10 patients (7 male, median age 48.5 years). The core temperature measured esophageally was 16.9–28.4 °C, median 22 °C. On admission 5 patients presented with asystole and 5 with ventricular fibrillation. Duration of circulatory arrest before ECMO implantation was 107 to 345 min (median 156 min). The duration of ECMO support was 1.5 to 91 h (median 22 h). Cardiorespiratory stability and full neurologic recovery was achieved in 7 patients. The duration of mechanical ventilation was 88–437 h (median 177 h) and the length of stay in the ICU was 8–26 days (median 15 days). All survivors had mildly impaired (1 patient, LVEF 40 %) or preserved (6 patients, LVEF 55–65 %) left ventricular systolic function at the time of discharge from ICU. The cause of death of non-survivors (three patients) was acute myocarditis, massive retroperitoneal bleeding, and massive gastrointestinal bleeding.

**Discussion and Conclusions:**

Our data confirm the high survival rate (70 %) and excellent neurologic outcome in hypothermic cardiac arrest. The following key elements seem to impact the final prognosis: the appropriate coordination of the rescue operation, immediate high-quality CPR (preferably using mechanical chest compression system) and application of ECMO for rewarming and cardiorespiratory support.

## Background

Accidental hypothermia is defined as an involuntary drop in core temperature below 35 °C. Recent guidelines recommend a standard classification of hypothermia based on a core temperature and vital signs (Swiss staging system). Severe hypothermia (core temperature of less than 28 °C) is associated with a high risk of circulatory arrest and high mortality (due to progressive systemic edema, respiratory insufficiency and neurologic complications) [1].

Currently, several non-invasive and invasive methods of rewarming are being used [1]. Successful management of severe accidental hypothermia has been achieved using invasive rewarming techniques in selected victims. Early rewarming with extracorporeal membrane oxygenation (ECMO) is recommended by the European Resuscitation Council as the method of choice in rewarming from severe accidental hypothermia with cardiac instability or circulatory arrest [2]. ECMO allows rapid and safe rewarming, enables continuous oxygenation and hemodynamic support. It decreases the incidence of severe cardiorespiratory instability that often complicates restoring normal body temperature [3].

In the years 2009–2013 the Polish National Statistics Department reported 2198 deaths due to exposure to excessive natural cold [4]. 624 of these patients (28.39 %) died in hospital. The introduction of innovative system for identification of hypothermic patients and early qualification for the extracorporeal rewarming may reduce the number of potentially preventable deaths. In July 2013 the Severe Accidental Hypothermia Center (SAHC) was founded in the John Paul II Hospital in Cracow [5]. It is a unit functioning within the structure of the Cardiac Surgery Clinic established in order to improve the effectiveness of the treatment of patients in severe hypothermia from the Lesser Poland (Małopolska) province (area of 15 100 square km, population of 3.3 million)

## Study objective

The purpose of the present study was to assess the outcome of patients with hypothermic circulatory arrest treated by means of arterio-venous extracorporeal membrane oxygenation (ECMO).

## Methods

The prospective observational case-series study was approved by the Local Ethical Committee of the John Paul II Hospital in Cracow. After full recovery, all the study subjects gave informed consent to the use of their medical data.

From 29 July 2013 to 29 November 2015, we consulted 137 hypothermic patients. 115 patients in the II and III stages of severe hypothermia (according to the Swiss scale i.e., stable circulation and respiration) were rewarmed using conventional methods and 22 patients were accepted for extracorporeal rewarming. 10 patients with cardiac arrest and 12 with severe cardiogenic shock and core temperature below 28 C were enrolled for veno arterial ECMO treatment.

All ten patients who were admitted to the Severe Accidental Hypothermia Center in hypothermic cardiac arrest were enrolled in the study. All of them fulfilled the established criteria and undergone rewarming with ECMO in arterio-venous configuration (Maquet Rotaflow). Surgical cannulation of femoral vessels was performed, preferably unilateral. Continuous unfractionated heparin infusion was administered to provide adequate level of anticoagulation, with ACT times being in the range of 140–160 s.

Blood samples were collected for the arterial gases, blood glucose and chemistry analysis at the time of admission and immediately after rewarming. Blood tests were assayed by routine automated laboratory techniques (Cobas System 600, Roche Diagnostics GmbH, Manheim, Germany). All biochemical analyses were performed in the central hospital laboratory, certified with a cardiac and clinical chemistry program by RIQAS (Randox Quality Assessment Scheme, UK). Transthoracic echocardiography was performed on discharge from the Intensive Care Unit (ICU) after achieving cardiovascular stability. The examinations were conducted by means of a portable ultrasound system equipped with a 1–5 MHz transthoracic phased-array transducer (CX 50, Philips, Eindhoven, Netherlands).

Since restoration of hemodynamic stability was the priority of extracorporeal rewarming, the targeted temperature was 36,6 °C – no post-cardiac arrest therapeutic hypothermia was maintained.

## Results

The study population consisted of 10 patients (7 male, age 25–78 years, median age 48.5 years), transported by ambulance (6 patients) or by helicopter (4 patients) from the distance 1–128 km (median 85.5 km). They were admitted in circulatory arrest secondary to hypothermia (core temperature measured esophageally 16.9–28.4 °C, median 22 °C). Demographic and admission clinical data are presented in Table 1. Five patients (50 %) were reported by the emergency medical services and were transported directly to Severe Accidental Hypothermia Center. The other five patients were transferred via regional emergency departments. It should be noticed, that at least three patients deteriorated rapidly during initial treatment (triage, examination or decontamination). On admission to SAHC 5 patients presented with asystole and 5 with ventricular fibrillation. Duration of circulatory arrest before ECMO implantation was 107 to 345 min (median 156 min). The duration of ECMO support was 1.5 to 91 h (median 22 h) (Table 2).

**Table 1:** Demographic and clinical characteristics of the study population on admission

Nr	Sex/age (yrs)	Type of accident	Transportation distance (km)	Type of transportation	Admission ECG	Core esophageal temperature (°C)
1	M/29	Alpine	98	Abmulance	Asystole	22.3
2	M/52	Urban	1	Ambulance	VF	22.2
3	M/78	Urban	73	Ambulance	VF	24
4	M/55	Urban	47	Ambulance	Asystole	24.1
5	F/25	Alpine (avalanche)	128	Helicopter	VF	16.9
6	M/68	Urban	102	Helicopter	VF	24
7	F/38	Urban	5	Ambulance	VF	25.4
8	M/45	Urban	114	Helicopter	Asystole	22
9	M54	Urban	102	Helicopter	Asystole	25.9
10	F/28	Diving	9	Ambulance	Asystole	28.4

**Table 2:** Hemodynamic data and clinical outcome of the study population

Nr	Duration of cardiac arrest before ECM implantation (min)	Rewarming rate (C/h)	Duration of ECMO rewarming (h)	Mechanical ventilation (h)	Duration of ICU stay (days)	Outcome	Discharge LVEF (%)	Clinical status at discharge from ICU
1	150	1.6	32	94	8	Fully recovered	65	GCS 15, CPC 1
2	140	4.0	22	88	11	Fully recovered	40	GCS 15, CPC 1
3	144	2.0	27	313	22	Fully recovered	60	GCS 15, CPC 1
4	155	0.5	23	319	15	Fully recocvered	55	GCS 15, CPC 1
5	345	4.5	91	134	26	Fully Recovered	60	GCS 15, CPC 1
6	177	2.0	8	177	12	Fully recovered	60	GCS 15, CPC 1
7	107	1.5	22	437	21	Fully recovered	60	GCS 15, CPC 1
8	280	3	5	5	1	Died	N/A	N/A
9	250	1.25	9	9	1	Died	N/A	N/A
10	157	4.0	1.5	1.5	0	Died	N/A	N/A

Analysis of arterial blood samples collected on admission revealed metabolic acidosis (pH 6.56–7.14, bicarbonate level 5.6–17.3 mmol/l) and elevated lactate levels (7.1–17 mmol/l) in all patients. Blood glucose analysis showed hyperglycemia (median 12 mmol/) in 7 patients, 2 patients were hypoglycemic (2.6 mmol/l and 2.8 mmol/l). Two patients were hypokalemic, 1 patient presented with hyperkalemia of 6.4 mmol/l, (Table 3). Arterial blood gases, blood glucose and chemistry results immediately after ECMO rewarming are summarized in Table 4.

**Table 3:** Arterial blood gases, chemistry and blood glucose level in the study population on admission

Nr	pH	pCO_2_ (mmHg)	pO_2_ (mmHg)	BE (mmol/l)	K (mmol/l)	Na (mmol/l)	Lactates (mmol/l)	HCO_3_ (mmol/l)	Glucose (mmol/l)	Hemoglobin (g/dl)
1	6.91	75.8	49.4	−20.8	4.6	141	13.5	14.4	2.8	14.5
2	6.89	94.3	47.1	−18.3	4.2	146	7.1	17.3	14.9	12.8
3	6.78	64.6	85.8	−24.7	3.6	142	12.8	9.1	2.6	8.9
4	7.14	43.1	69.8	−14.3	3.1	135	7.6	14.1	9.4	12.7
5	6.64	79	92	−33.2	4.3	139	17	7.9	9	14.3
6	6.95	73.9	53.8	−20.3	3.7	146	10.6	15.3	12	16.7
7	6.94	43.3	138	−22.2	2	128	12	8.9	28	9.6
8	6.99	61.2	48.1	−18.3	5	143	11,9	14.1	6.3	13.6
9	6.67	57.4	48.8	−31.2	4.4	152	16	7.6	7	15.6
10	6.56	65.8	156	−34.6	6.4	131	14	5.6	33	13

**Table 4:** Arterial blood gases, chemistry and blood glucose level in the survivors immediately after ECMO rewarming

Nr	pH	pCO_2_ (mmHg)	pO_2_ (mmHg)	BE (mmol/l)	K (mmol/l)	Na (mmol/l)	Lactates (mmol/l)	HCO_3_ (mmol/l)	Glucose (mmol/l)	Hemoglobin (g/dl)
1	7.52	27.1	153	−0.1	3	146	2.3	21.8	3.3	10.2
2	7.25	30.2	142	−13.1	3.7	147	8,7	12.7	6.6	9.1
3	7.08	25.9	137	−21.4	4	143	17	7.3	9.1	10.1
4	7.31	41.1	79.1	−3.3	4.7	136	1.8	21.6	9.1	9.1
5	7.19	40.9	24,3	−11.8	3.8	146	7.5	15	6.2	7.3
6	7.17	28.5	162	−17.5	3.6	150	14	9.9	8.6	12
7	7.39	31.1	235	−5.5	4.7	140	8.9	18.4	6.4	7.9

One patient (a 28-year-old female, injured while diving) died in the operating theatre, 1.5 h after ECMO implantation. Autopsy revealed active myocarditis. The other two patients died in the intensive care unit several hours after ECMO implantation. One of them died due to massive retroperitoneal bleeding, in a second one autopsy showed massive gastrointestinal bleeding.

Cardiorespiratory stability and full neurologic recovery was achieved in 7 patients (Cerebral Performance Category 1). The duration of mechanical ventilation was 88–437 h (median 177 h) and the length of stay in the ICU was 8–26 days (median 15 days). All survivors had mildly impaired (1 patient, LVEF 40 %) or preserved (6 patients, LVEF 55–65 %) left ventricular systolic function at the time of discharge from ICU (Table 2).

## Discussion

Severe accidental hypothermia is a rare condition associated with significant morbidity and mortality [1]. Severe Accidental Hypothermia Center provides 24 h on-call consultation and qualification for extracorporeal rewarming. A trilateral phone calls between the dispatch center, on-site emergency medical team and the hypothermia coordinator are possible enabling immediate decision making. A crucial issue is precise coordination of rescue achieved with localization system and close cooperation with Dispatch Centre. Planning and proceeding in accordance with guidelines and dedicated protocols, enables shortening the transport time and implementing an effective treatment. In our study group, there were seven patients transported from a long distance (47–128 km) - four patients by helicopter and three by ambulance.

Prognosis in hypothermic circulatory arrest is surprisingly good [3]. This is most probably associated with protecting effects of hypothermia itself. Even prolonged cardiac arrest and prolonged cardio-pulmonary resuscitation (CPR) do not usually cause hypoxic brain damage in severe hypothermia. There are several reports of full neurologic recovery of patients with hypothermic circulatory arrest [3]. According to the recent guidelines, in patients with severe hypothermia, CPR can be delayed and intermittent if it is not possible to perform continuous CPR (for example during difficult evacuations). However, it is postulated to minimize interruptions and apply mechanical chest compressions as soon as possible [6]. Mechanical compression has not been shown to have a survival advantage over manual compression, but the latter cannot be done effectively in a moving ambulance. Thus, the immediate provision of the device for mechanical chest compression is very important in carrying out the rescue operation. The Severe Accidental Hypothermia Center created a regularly updated map of such equipment in local emergency medicine system. In all patients in our study group, a high-quality CPR was initiated immediately (including endotrachaeal intubation, ventilation, infusion of inotropes/vasopressors) and performed continuously with short interruptions. In all 10 cases mechanical chest compression devices were used.

The survival rate in our study population was 70 % that is consistent with the available reports. There must be highlighted that all survivors left ICU in excellent cardiovascular (LVEF 40-65 %) and neurological status (CPC 1). We did not observe any complications associated with ECMO implantation or application (the longest duration of ECMO use was 91 h). Wanscher et al. reported a 100 % survival (with a full neurologic recovery in 85.7 % of patients) in 7 victims of a boat accident with hypothermic cardiac arrest, treated with ECMO rewarming [7]. Ruttman et al. in the retrospective comparative study of 59 patients following hypothermic cardiac arrest noted a significant survival benefit with ECMO (75 vs 34 %) [8]. Mair et al. conducted a retrospective observational study of 22 patients following hypothermic cardiac arrest [9]. Overall survival was 45.5 %. Walpoth et al. showed a complete recovery in 93.3 % of 15 long-term survivors of deep hypothermic cardiac arrest [10]. Eich et al. conducted a retrospective comparative study of 12 patients following drowning and hypothermic cardiac arrest treated with CBP (cardio-pulmonary bypass). Survival was only 41,7 % most probably due to asphyxia. Comparison between survivors and non-survivors revealed low submersion times, early initiation of basic life support, hypokalemia, female gender and slow rewarming to be significant prognostic indicators of favorable outcomes [11]. Debaty et al. performed a retrospective cohort study on 48 patients with accidental hypothermia in French Alps over a 10-year period. Overall mortality was 50 %, and cardiac arrest related to rescue collapse was associated with favorable outcome [12].

## Conclusion

Our data confirm the high survival rate (70 %) and excellent neurologic outcome after cardiac arrest secondary to severe accidental hypothermia. The following key elements seem to impact the final prognosis: the appropriate coordination of the rescue operation, immediate high-quality CPR (preferably using mechanical chest compression system) and application of ECMO for rewarming and cardiorespiratory support

## Abbreviations

CPB, cardio-pulmonary bypass; CPC, cerebral performance category; CPR, cardio-pulmonary resuscitation; ECMO, arterio-venous extracorporeal membrane oxygenation; ICU, intensive care unit; LVEF, left ventricular ejection fraction; SAHC, Severe Accidental Hypothermia Centre
